# Comment on 'D-dimer and high-sensitivity C-reactive protein levels to predict venous thromboembolism recurrence after discontinuation of anticoagulation for cancer-associated thrombosis'

**DOI:** 10.1038/s41416-018-0361-x

**Published:** 2019-01-30

**Authors:** Frederikus A. Klok, Henri H. Versteeg, Arie J. Verschoor, Menno V. Huisman

**Affiliations:** 1 Department of Thrombosis and Haemostasis, Leiden University Medical Center, Leiden, The Netherlands; 2 Department of Medical Oncology, Leiden University Medical Centre, Leiden, The Netherlands

**Keywords:** Outcomes research, Cancer

With great interest, we read the paper by Jara-Palomares and colleagues, in which they show that D-dimer and high-sensitivity C-reactive protein predict recurrent venous thromboembolism (VTE) after discontinuation of anticoagulant treatment for cancer-associated VTE.^1^ Indeed, these results provide ground for discussion on a personalised approach to anticoagulation duration in this patient category, especially because of the well-known high risk of bleeding in anticoagulated patients with cancer.^2^

As an explanation for their findings, the authors propose that the biomarkers under study are markers of global coagulation activation, and thus of the potential for pathological clot formation.^1^ We propose an additional hypothesis—D-dimer and high-sensitivity C-reactive protein are not only markers of haemostatic activity in cancer patients, but also of active cancer itself.^3,4^ In this study, more than half of the patients had non-metastatic disease, and short life expectancy, cerebral metastases and a subjective assessment of the treating physician ('absence of circumstances favouring treatment maintenance based on the clinician’s discretion') were the exclusion criteria. Therefore, it seems that discontinuation of anticoagulation was not considered in patients with advanced cancer. Moreover, we wonder whether the cancer was in remission in a relevant number of the 114 included patients, and especially in those with low levels of the biomarkers.

Our group has previously shown that patients cured of cancer could safely stop anticoagulants with a low risk of recurrent VTE.^5^ The incidence rate of recurrent VTE in these particular patients was 3.2 per 100 patient-years (95% confidence interval 1.5–5.9). Notably, 70% of patients with recurrent VTE experienced a cancer relapse shortly before or after this recurrent VTE event.^5^

Because poor performance status and metastasized cancer were predictors of recurrent VTE in the study by Jara-Palomares and colleagues, (active) cancer status at the moment of inclusion may prove to be a relevant determinant of their main outcome.^1^ It would therefore be of great value, and would put their results in another perspective, if the authors could present their main results stratified for cancer status at inclusion.
